# Association between Epicardial Adipose Tissue and Atrial Fibrillation in Patients with Transfusion-Dependent β-Thalassemia

**DOI:** 10.3390/jcm13123471

**Published:** 2024-06-14

**Authors:** Michele Malagù, Elisabetta Tonet, Giovanni Orazio, Filomena Longo, Martina De Raffele, Paolo Sirugo, Andrea Capanni, Stefano Clò, Maria Letizia Berloni, Federico Marchini, Marco Manfrini, Elisa Mari, Olga Soffritti, Martina Culcasi, Cristina Balla, Francesco Vitali, Alberto Cossu, Matteo Bertini

**Affiliations:** 1 Cardiology Unit, Azienda Ospedaliero-Universitaria di Ferrara, 44124 Ferrara, Italy; 2 Day Hospital Thalassemia and Hemoglobinopathies, Azienda Ospedaliero-Universitaria di Ferrara, 44124 Ferrara, Italy; 3 Department of Medical Sciences, Centre for Clinical and Epidemiological Research, University of Ferrara, 44121 Ferrara, Italy; 4 Radiology Unit, Azienda Ospedaliero-Universitaria di Ferrara, 44124 Ferrara, Italy

**Keywords:** thalassemia, hemoglobinopathy, epicardial fat, atrial fibrillation, arrhythmia, prediction, stroke, echocardiography, magnetic resonance, imaging

## Abstract

**Background:** Modern treatments for transfusion-dependent β-thalassemia (TDβT) have allowed patients to reach high life expectancy with no iron overload. Despite survival improvement, atrial fibrillation (AF) has emerged as a relevant issue. AF pathophysiology and characteristics in TDβT are different than in the general population. Epicardial adipose tissue (EAT) may play a role but its relationship with AF in patients with TDβT has not been explored. **Methods:** A monocentric, cross-sectional study, enrolling consecutive patients with TDβT. Epicardial adipose tissue (EAT) was evaluated at magnetic resonance. Characteristics of patients with and without history of AF were investigated. Factors independently associated with AF prevalence were analyzed. **Results:** A total of 116 patients were enrolled. All patients were treated with regular chelation therapy. The prevalence of AF was 29.3% (34/116). Cardiac T2* and liver iron concentration were no different between patients with and without AF. EAT thickness was significantly higher in patients with AF at left atrium, right atrium and right ventricle (5.0 vs. 4.0 mm, *p* < 0.01, 4.4 vs. 4.0, *p* = 0.02 and 5.0 vs. 4.3, *p* = 0.04). Patients with AF presented with older age, (53 vs. 49 years, *p* < 0.01), more hypothyroidism (44.1 vs. 20.7%, *p* = 0.01), pulmonary hypertension (23.5 vs. 2.4% *p* < 0.01), splenectomy (88.2 vs. 64.6%, *p* = 0.01), higher right and left atrial volume (61 vs. 40 and 74 vs. 43 mL, both *p* < 0.01). At multivariable analysis, hypothyroidism, left atrial volume and left atrial EAT were independently associated with AF (odds ratio 9.95, 1.09 and 1.91, respectively). **Conclusions:** In a contemporary cohort of patients with TDβT, treated with regular chelation therapy, prevalence of AF was unrelated to iron overload. EAT was independently associated with AF.

## 1. Introduction

β-thalassemia is an inherited disease due to reduced or absent production of hemoglobin [1]. Patients affected by β-thalassemia are commonly treated with lifelong red blood cell transfusions and iron chelation therapy [2]. Nowadays, a prolonged life expectancy is possible but several comorbidities may develop, among which atrial fibrillation (AF) represents a frequent issue [3,4]. The prevalence of AF in patients with β-thalassemia is higher than in the general population due to a complex pathophysiology resulting from the combination of chronic anemia, blood transfusions, iron overload, reactive oxygen species, chronically elevated cardiac output, inflammation, atrial fibrosis, neuroendocrine imbalance and metabolic disorders [3]. The role of iron in the development of AF has been questioned [5]. Additionally, not all patients treated for β-thalassemia develop AF. The analysis of other potential risk factors could help to investigate this emerging problem. In recent years, epicardial adipose tissue (EAT) emerged as a novel marker of AF in the general population. EAT is a fat tissue depot located between the myocardium and the visceral pericardium [6]. Previous studies in the general population showed that EAT is associated with AF prevalence, independently from other risk factors or obesity, both for paroxysmal and persistent AF [7,8]. Left atrial EAT can lead to AF for the secretion of factors determining fibrosis, inflammation, free fatty acid infiltration and autonomic imbalance [9]. Nowadays, EAT is a recognized risk factor for AF and represents a rapidly growing research field, provoking great interest [9]. The interesting relationship between β-thalassemia and AF raises the question as to whether AF may be associated with EAT, even in this particular subset of patients. Considering that the mechanisms leading to AF in patients with thalassemia may be different than those in the general population, the question deserves proper investigation. To date, the role of EAT in patients with β-thalassemia has not been explored. A better understanding of this relationship could provide more evidence regarding a complex pathophysiology and may have potential implications in risk stratification, leading to improved clinical management.

## 2. Methods

The main objective of this study was to evaluate the association between EAT and AF in a contemporary cohort of patients with transfusion-dependent β-thalassemia (TDβT), treated with regular chelation therapy. Other objectives were to assess the prevalence of AF in this population, to describe AF characteristics and to explore the relationship between AF and other clinical, imaging, and laboratory parameters.

Patients were enrolled at the Azienda Ospedaliero-Universitaria di Ferrara, Italy. Inclusion criteria were as follows: TDβT, age > 18 years. Exclusion criteria were as follows: state of pregnancy, inability to give informed consent, previous cardiac surgery. The present study is a cross-sectional analysis. This analysis is part of the project “*Atrial fibrillation in β-thalassemia*”. This study was registered on ClinicalTrials.gov (NCT05508932). Study protocol was approved by the local Ethics Committee (Comitato Etico Indipendente di Area Vasta Emilia Centro) and all patients signed informed consent.

Patients underwent routine cardiological examination, ECG and echocardiography as part of the standard of care. AF diagnosis was made at 12-lead ECG according to European Society of Cardiology guidelines [10]. When present, history of AF diagnosed at previous ECGs was considered.

**Figure 1:**
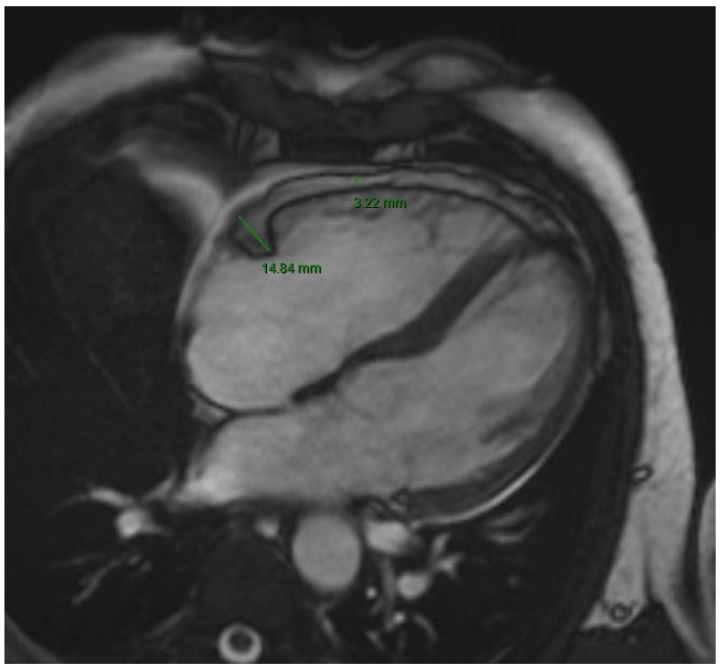
Epicardial adipose tissue evaluation at cardiac magnetic resonance.

All patients underwent a 1.5 T cardiac magnetic resonance (CMR) in the setting of iron overload monitoring. CMR (Siemens, Magnetom Aera) protocol included steady state free precession (SSFP) sequences and T2* imaging. According to data from the literature, EAT thickness was assessed using SSFP sequences because of their ability to distinguish the fat from the muscle and the blood, providing good quality in the EAT evaluation [11,12]. EAT was measured in the following locations: right ventricular free wall, right atrial free wall, right atrioventricular groove, left ventricular free wall, left atrial free wall, left atrioventricular groove (Figure 1). Transversal four chamber view was the most used for EAT measure, followed by short axis views. The investigators performing EAT measurements were blinded to the medical records of the patients and to the history of AF.

Figure 1 shows thickness of epicardial adipose tissue measured at right atrioventricular groove (14.84 mm) and right ventricular free wall (3.22 mm).

Data regarding medical history, laboratory tests, imaging and medical therapy were collected. Heart failure history was defined as left ventricular ejection fraction <40% or previous hospitalization for heart failure. Liver iron concentration was assessed with magnetic resonance imaging.

Baseline characteristics were summarized as frequencies and percentage for categorical variables and as median and interquartile range for continuous variables. Differences between groups were analyzed using the Pearson χ^2^ test and the Mann–Whitney U test where appropriate. Univariate and multivariable analysis was performed. Statistical analyses were performed with SPSS version 25 (IBM Corporation, Armonk, NY, USA).

## 3. Results

A total of 116 patients were enrolled in this study. The AF prevalence was 29.3% (34/116 patients). ECG and echocardiography were performed less than 6 months before the enrollment of all patients. Baseline characteristics of study population are shown in Table 1.

No patient had liver cirrhosis. No patient had creatinine clearance <30 mL/min. As chelation therapy, 43 patients were treated with deferoxamine (37.1%), 46 with deferasirox (39.7%), and 31 with deferiprone (26.7%). Several differences were found between the study groups. Compared to patients with no history of AF, patients with AF were older (median 53 vs. 49 years, *p* < 0.01) and showed higher prevalence of hypothyroidism (44.1 vs. 20.7%, *p* = 0.01), pulmonary hypertension (23.5 vs. 2.4%, *p* < 0.01), and splenectomy (88.2 vs. 64.6%, *p* = 0.01).

Serum ferritin was lower in patients with history of AF (median 342 vs. 608 ng/mL, *p* < 0.01). Left ventricular ejection fraction resulted normal in both groups but slightly higher in patients with no history of AF (median 61 vs. 60%, *p* = 0.01). Left and right atrial volume were significantly higher in patients with AF than in patients with no AF (median 74 vs. 43 mL, *p* < 0.01 and 61 vs. 40 mL, *p* < 0.01, respectively).

Patients with AF showed significantly higher EAT in different locations: the right atrial free wall (median 4.4 vs. 4.0 mm, *p* = 0.02), right ventricular free wall (5.0 vs. 4.3 mm, *p* = 0.04), and left atrial free wall (5.0 vs. 4.0 mm, *p* < 0.01). In the right atrio-ventricular groove, left atrio-ventricular groove, and left ventricle, EAT was not different between the two groups.

The characteristics of AF patients are shown in Table 2.

The most common type was paroxysmal (21 subjects, 61.8%). The CHA_2_DS_2_VASc score was 0 or 1 in 23 patients (67.7%). Other supraventricular arrhythmias had been reported in 21 patients (61.8%).

At univariate analysis, age, hypothyroidism, splenectomy, LA volume, diastolic dysfunction and LA EAT were significantly associated with AF. At multivariate analysis, only hypothyroidism, LA volume and LA EAT were independently associated with AF (Table 3), with an area under the curve (AUC) equals to 0.87 (95% confidence interval 0.79–0.96, *p* < 0.01).

## 4. Discussion

**Figure 2:**
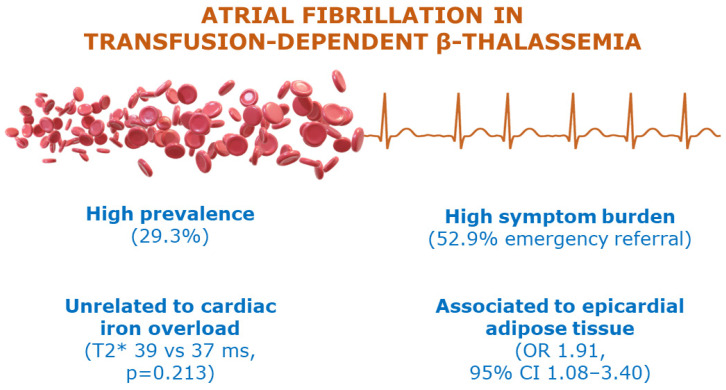
Central illustration.

The main result of our study was that EAT, and not iron overload, was independently related to AF in TDβT patients. LA volume and hypothyroidism were also independently associated to AF. Other relevant findings concern the prevalence of AF and its characteristics (Figure 2).

The prevalence of AF in our cohort was 29.3%. While, in the general population, AF affects 2–4% of subjects, it is known that this condition is much more prevalent in thalassemia [10]. Previous studies in cohorts of patients with β-thalassemia reported AF prevalence ranging from 3 to 26% [13,14,15,16]. However, those studies were heterogeneous, with AF epidemiology not being the main endpoint and showing differences in age and iron overload status. Our study cohort presented advanced age (median age 50 years, which may be considered elder for thalassemic patients but, on the other hand, is considerably young for AF), optimal chelation status (median liver iron concentration 2.77 mg Fe/g) and no signs of cardiac iron overload (median T2* 39.0 ms). The outstanding chelation regimen was confirmed by the low risk of heart failure (median EF 60%). Therefore, our data showed the high prevalence of AF in patients with TDβT and suggest that once thalassemic patients are optimally treated, they may reach advanced age with no overt heart failure but a high rate of AF. Our center blazed a trail in regards to the initiation of a routine chelation regimen decades ago. With the implementation of optimal chelation therapy all around the world, the issue of AF will probably become more and more topical in the near future.

No differences in T2* were reported between patients with and without AF (39.0 vs. 37.0 ms, *p* = 0.213). In the past, evidence was given that supraventricular arrhythmias were associated to cardiac iron overload [15,17]. However, prospective studies specifically investigating the relationship between arrhythmias and iron in contemporary cohorts, found that AF was not related to iron overload [5]. Our data confirm that the pathogenesis of AF in patients with TDβT may be unrelated to iron overload. This observation leads to the need of further investigations on the pathophysiological mechanism of AF in those patients. We could only hypothesize a fundamental role of anemia, hyperdynamic circulation, inflammation and fibrosis but further studies are warranted.

The main aim of our analysis was to investigate the relationship between AF and EAT. Our results showed significantly higher EAT thickness in different locations (right atrium, left atrium, right ventricle free wall) in thalassemic patients with AF, compared to thalassemic patients without AF. Multivariable analysis confirmed an independent association between EAT and AF. From previous observations, it was known that, in the general population, EAT is independently associated with AF [7,8]. However, the pathophysiology of AF in patients with β-thalassemia is peculiar and different from the general population, even in the presence of common risk factors. Our findings suggest that the independent association between EAT and AF may be true also for patients with β-thalassemia. However, no causal relationship is known. Longitudinal studies could help to better understand this association. A possible explanation is that EAT could potentially trigger local cardiac inflammation that may promote the development of AF in those patients. Recent evidence showed that the degree of inflammation in adipose tissue can be non-invasively evaluated using cardiac CT [18]. In the presence of inflammation of adipose tissue, adipogenesis result inhibited in favor of lipolysis and water content in adipocytes increases. This process alters the overall attenuation of EAT on cardiac CT due to edema [18]. Further studies could provide additional insights.

When present, AF was more often paroxysmal or persistent. Most patients have previous emergency room referral for AF (52.9%) and have undergone several treatments for rhythm control such as electrical cardioversion (23.5%), pharmacological cardioversion (14.7%) and even transcatheter ablation (29.4%). These findings suggest that thalassemic patients are highly symptomatic and have poor tolerance to AF. Patients with thalassemia usually live in a balance between young age, active lifestyle and chronic anemia, periodic blood transfusions, and comorbidities. We can hypothesize that AF may disrupt this balance and lead to further medical treatments. The efficacy of rhythm control strategies in those patients should be evaluated with further studies.

Despite the low CHA_2_DS_2_VASc score in our cohort, 2.6% of patients had a previous history of ischemic stroke. Whether the stroke was cardioembolic was not established. However, it is well known that patients with β-thalassemia have a higher incidence of thromboembolic events compared to the general population and the higher incidence of AF may play a role [19,20]. Anticoagulant therapy is effective in reducing thromboembolic events in patients with AF. Among patients with β-thalassemia and AF, in which chronic anemia, hepatic comorbidities, and splenectomy raised concerns of hemorrhagic complications, the use non-vitamin K oral anticoagulants has been described in small cohorts and seems safe and effective [21,22].

## 5. Limitations

This was a single-center cross-sectional study. No insights into pathophysiological mechanisms or causative relationships are given. However, our aim was to describe the characteristics of a specific population and generate hypotheses for further studies.

## 6. Conclusions

The prevalence of AF in patients with TDβT is high. AF is not related to iron overload in optimally treated patients with modern chelation regimens. Hypothyroidism, LA volume, and LA EAT are independently associated with AF in patients with TDβT.

## Figures and Tables

**Table 1:** Patient characteristics.

Variable	Study Population (*n* = 116)	No AF (*n* = 82)	AF (*n* = 34)	*p* Value
Male sex	60 (51.7%)	39 (47.6%)	21 (61.8%)	0.22
Age (years)	50 [45–55]	49 [43–54]	53 [48–57]	**<0.01**
BMI (kg/m^2^)	22.1 [20.6–24.2]	22.1 [20.5–24.2]	22.4 [20.8–24.0]	0.96
Arterial hypertension	13 (11.2%)	7 (8.5%)	6 (17.6%)	0.20
Dyslipidemia	2 (1.7%)	2 (2.4%)	0 (0%)	1.00
Smoking habit				0.91
- Active	16 (13.8%)	12 (14.6%)	4 (11.8%)	
- Previous	21 (18.1%)	14 (17.1%)	7 (20.6%)	
Altered glucose metabolism				0.29
- Impaired fasting glucose	2 (1.7%)	2 (2.4%)	0 (0%)	
- Abnormal OGTT	3 (2.6%)	1 (1.2%)	2 (5.9%)	
- Diabetes mellitus	24 (20.7%)	19 (23.2%)	5 (14.7%)	
Hyperinsulinism	1 (0.9%)	0 (0%)	1 (2.9%)	0.29
Hypothyroidism	32 (27.6%)	17 (20.7%)	15 (44.1%)	**0.01**
Hyperthyroidism	1 (0.9%)	0 (0%)	1 (2.9%)	0.29
Hypoparathyroidism	4 (3.4%)	2 (2.4%)	2 (5.9%)	0.29
GH deficiency	4 (3.4%)	3 (3.7%)	1 (2.9%)	1.00
Hypogonadism	33 (28.4%)	21 (25.6%)	12 (35.3%)	0.37
Osteoporosis	65 (56.0%)	46 (56.1%)	19 (55.9%)	1.00
Ischemic stroke	3 (2.6%)	2 (2.4%)	1 (2.9%)	1.00
Pulmonary hypertension	10 (8.6%)	2 (2.4%)	8 (23.5%)	**<0.01**
Splenectomy	83 (71.6%)	53 (64.6%)	30 (88.2%)	**0.01**
COPD	2 (1.7%)	1 (1.2%)	1 (2.9%)	0.50
LIC (mg Fe/g)	2.77 [1.39–5.38]	2.85 [1.56–5.88]	1.84 [1.31–4.03]	0.13
Heart failure	5 (4.3%)	3 (3.7%)	2 (5.9%)	0.63
WBC (×10^3^/µL)	9.5 [7.3–12.9]	9.6 [6.7–13.1]	9.4 [7.9–11.6]	0.67
Frequency of transfusions (days)	15 [15–21]	15 [15–21]	15 [15–20]	0.32
Pre-transfusion Hb (g/dL)	9.9 [9.4–10.4]	9.8 [9.4–10.3]	10.0 [9.3–10.6]	0.28
Platelets (×10^3^/µL)	446 [296–571]	437 [292–583]	454 [390–560]	0.79
Ferritin (ng/mL)	531 [325–782]	608 [397–866]	342 [245–595]	**<0.01**
Soluble transferrin receptor (mg/dL)	3.3 [2.6–4.6]	3.4 [2.6–4.6]	3.2 [2.4–3.9]	0.43
Heart rate (bpm)	72 [65–79]	72 [65–79]	74 [64–79]	0.96
P wave duration (msec)	85 [75–96]	85 [75–96]	90 [77–100]	0.24
QTc interval, Bazett (msec)	414 [394–433]	414 [395–429]	421 [392–438]	0.44
LV EDV index (mL/m^2^)	61.7 [54.1–72.1]	62.2 [55.5–73.6]	61.6 [53.3–66.2]	0.34
Ejection fraction (%)	60.0 [57.0–65.0]	61.0 [60.0–65.0]	60.0 [55.0–61.5]	**0.01**
Interventricular septum thickness (mm)	0.9 [0.8–1.0]	0.9 [0.8–1.0]	0.9 [0.8–1.1]	0.24
LA volume (mL)	49 [35–67]	43 [33–55]	74 [59–88]	**<0.01**
RA volume (mL)	45 [32–58]	40 [26–48]	61 [49–81]	**<0.01**
Diastolic dysfunction	20 (21.3%)	11 (15.9%)	9 (36%)	**<0.01**
TAPSE (cm)	2.4 [2.2–2.7]	2.4 [2.2–2.7]	2.4 [2.3–3.2]	0.36
T2* (ms)	39.0 [35.0–41.0]	39.0 [36.0–41.3]	37.0 [33.5–41.0]	0.21
RA EAT (mm)	4.0 [3.3–4.7]	4.0 [3.2–4.4]	4.4 [3.9–5.0]	**0.02**
RAVG EAT (mm)	13.0 [11.2–15.0]	13.1 [11.7–14.7]	12.2 [10.5–16.1]	0.69
RV EAT (mm)	4.6 [3.6–5.6]	4.3 [3.6–5.5]	5.0 [4.4–6.2]	**0.04**
LA EAT (mm)	4.4 [3.6–5.5]	4.0 [3.4–5.2]	5.0 [4.2–6.0]	**<0.01**
LAVG EAT (mm)	12.4 [10.7–13.7]	12.3 [10.8–13.5]	12.6 [9.7–14.8]	0.64
LV EAT (mm)	4.4 [3.3–5.2]	4.3 [3.2–5.3]	4.5 [3.7–4.8]	0.79

Data are expressed as number (percentage) or median [interquartile range]. BMI: body mass index; OGTT: oral glucose tolerance test; GH: growth hormone; COPD: chronic obstructive pulmonary disease; LIC: liver iron concentration; WBC: white blood cells; Hb: hemoglobin; LV EDV: left ventricular end diastolic volume; LA: left atrium; RA: right atrium; TAPSE: tricuspid annular plane systolic excursion; RA EAT: right atrial epicardial adipose tissue; RAVG EAT: right atrioventricular groove epicardial adipose tissue; RV EAT: right ventricular epicardial adipose tissue; LA EAT: left atrial epicardial adipose tissue; LAVG EAT: left atrioventricular groove epicardial adipose tissue; LV EAT: left ventricular epicardial adipose tissue. Statistically significant *p* values are shown in bold.

**Table 2:** Characteristics of atrial fibrillation.

Variable	Patients with AF (*n* = 34)
AF type	
- Paroxysmal	21 (61.8%)
- Persistent	7 (20.6%)
- Permanent	6 (17.6%)
CHA_2_DS_2_VASc score	
- 0	14 (41.2%)
- 1	9 (26.5%)
- 2	8 (23.5%)
- 3	2 (5.9%)
- 4	1 (2.9%)
- >4	0 (0%)
AF ablation	10 (29.4%)
Cardioversion	22 (64.7%)
- Electrical	8 (23.5%)
- Pharmacological	5 (14.7%)
- Both	9 (26.5%)
At least one emergency room referral for AF	18 (52.9%)
Number of AF episodes within last year	
- 0	23 (67.6%)
- 1	7 (20.6%)
- 2	1 (2.9%)
- >2	3 (8.8%)
EHRA symptom scale	
- 1	21 (61.8%)
- 2a	8 (23.5%)
- 2b	2 (5.9%)
- 3	3 (8.8%)
Other arrhythmias	21 (61.8%)
- Typical atrial flutter	6 (17.6%)
- Atypical atrial flutter	7 (20.6%)
- Paroxysmal SVT	2 (5.9%)
- Atrial tachycardia	5 (14.7%)

AF: atrial fibrillation; EHRA: European heart rhythm association; SVT: supraventricular tachycardia.

**Table 3:** Univariate and multivariable analysis.

Variable	Univariate Analysis			Multivariable Analysis		
	Odds Ratio	95% CI	*p* Value	Odds Ratio	95% CI	*p* Value
Age	1.05	1.00–1.10	**0.04**			
Hypothyroidism	3.02	1.28–7.15	**0.01**	9.95	1.99–49.85	**<0.01**
Splenectomy	4.10	1.32–12.80	**0.02**			
LA volume	1.04	1.02–1.07	**<0.01**	1.09	1.03–1.15	**<0.01**
Diastolic dysfunction	3.39	1.18–9.75	**0.02**			
LA EAT	1.61	1.14–2.28	**0.01**	1.91	1.08–3.40	**0.01**

CI: confidence interval; LA: left atrium, EAT, epicardial adipose tissue. Statistically significant *p* values are shown in bold.

## Data Availability

The data underlying this article will be shared on reasonable request to the corresponding author.
